# Assessment of Transportation by Air for Patients with Acute ST-Elevation Myocardial Infarction from Non-PCI Centers

**DOI:** 10.3390/healthcare9030299

**Published:** 2021-03-08

**Authors:** Angel Liviu Trifan, Liliana Dragomir, Mihaela Anghele, Eva Maria Elkan, Sorina Munteanu, Cosmina Moscu, Valerian-Ionuț Stoian, Anca Telehuz, Monica Laura Zlati, Mihaiela Lungu, Doina Carina Voinescu, Diana Carmen Cimpoesu, Gabriela Stoleriu, Ion Sandu, Violeta Sapira

**Affiliations:** 1 Medical Department, Faculty of Medicine and Pharmacy, “Dunarea de Jos” University of Galati, 800008 Galati, Romania; trifanangel@gmail.com (A.L.T.); pletea.lili@yahoo.com (L.D.); anghele.mihaela@yahoo.com (M.A.); cojocarumariaeva@yahoo.com (E.M.E.); sorinanicoleta.munteanu@yahoo.com (S.M.); cosmina_caluian@yahoo.com (C.M.); valeryss17@gmail.com (V.-I.S.); ciosy@yahoo.com (A.T.); carinavoinescu@gmail.com (D.C.V.); violetasapira@yahoo.com (V.S.); 2 Research Center in the Field of Social Sciences, “Stefan cel Mare” University, 720229 Suceava, Romania; sorici.monica@usm.ro; 3 Emergency Department, Faculty of Medicine, “Grigore T. Popa” University of Medicine and Pharmacy, 700115 Iasi, Romania; dcimpoiesu@yahoo.com; 4 Derma Clinique, 121 Pacurari Street, 700523 Iasi, Romania; 5 Academy of Romanian Scientists (AOSR), 050094 Bucharest, Romania; ion.sandu@uaic.ro; 6 ARHEOINVEST Centrum, Institute of Interdisciplinary Research, “Alexandru Ioan Cuza” University of Iasi, 700506 Iasi, Romania; 7 Romanian Inventors Forum, 3 Sf. P. Movila Street, 700089 Iasi, Romania

**Keywords:** helicopter emergency medical service, percutaneous coronary intervention, ST-segment elevation myocardial infarction, ground emergency medical systems

## Abstract

The aim of this study was to assess the delays that can potentially occur in the emergency transfer of patients with ST-elevation myocardial infarction (STEMI) to percutaneous coronary intervention (PCI) centers. We conducted a retrospective study using the medical reports pertaining to 97 patients who presented to the Emergency Department of the Emergency County Hospital of Galati during the year of 2018 with the diagnosis of STEMI and meeting eligibility criteria for PCI, thus warranting transfer to a hospital with PCI facilities. The pick-up time of patients diagnosed with acute myocardial infarction from the emergency department by the transfer crew is significantly shorter (*p* < 0.05) than those transferred by air, regardless of the PCI center to which the transfer was performed, Iasi or Bucharest, when compared to the time required to process the patients transferred by land to the same PCI centers. The results of the study shows that the helicopter use for transferring acute myocardial infarction patients to a PCI center must be considered, given the distance between non-PCI and PCI centers is over 200 km.

## 1. Introduction

Primary percutaneous coronary intervention (PCI) constitutes the treatment of choice for patients with ST-segment elevation myocardial infarction (STEMI) [1]. Considering a similar treatment delay, randomized clinical trials in experienced centers have shown that primary PCI is superior to fibrinolysis in reducing mortality, reinfarction or stroke [1,2,3]. However, in certain circumstances, when primary PCI cannot be performed within the first 120 min of STEMI diagnosis, fibrinolysis should be performed per primam. Current guidelines recommend the interval between the first medical contact and the primary PCI to be 90 min or less [4], as any further delays may reduce the PCI benefits. Continuous planning advances in the emergency medical services and rapid transfer of STEMI patients to a center capable of percutaneous coronary intervention (PCI) are of paramount importance in minimizing timeframes.

There is also a general consensus that the primary PCI strategy [4] should also be performed in patients with symptoms lasting over 12 h if they show: ECG signs of persistent ischemia; persistent or recurrent pain and dynamic ECG changes and persistent or recurrent pain, symptoms and signs of heart failure, shock, or malignant arrhythmias.

In consequence, patients should be transported to a PCI-enabled facility as soon as possible after the administration of the fibrinolytic bolus. Given that at rescue, angioplasty is indicated in cases of fibrinolysis failure (e.g., decrease in ST segment elevation by less than 50% in the first 60–90 min after fibrinolytic administration) or in the presence of hemodynamic or electrical instability, worsening of ischemia or persistent chest pain [5,6], while the routine PCI strategy is also indicated after successful fibrinolysis (preferably 2–24 h after fibrinolysis) [7,8], it is recommended that STEMI patients should get to a center where PCI can be performed as soon as possible.

Any interventions attempting to reduce the time spent from diagnosis to PCI should be embraced. These include information exchange with the catheterization center via telephone with the timely preparation of a team, a clear chain of communication between the two health units, and simplified interhospital transfer processes and assessment of the fastest way to transfer the patient from the non-PCI center to the center where the PCI can be performed.

Therefore, helicopter emergency medical services (HEMS) and ground emergency medical services (GEMS) are an integral part of the transportation system used for patients with ST-elevation acute myocardial infarction (STEMI) to centers that can perform primary percutaneous coronary intervention (PCI).

From Galati, a non-PCI center, the transfer of STEMI patients to a center capable of PCI can be performed either through ground emergency medical systems (GEMS) or through helicopter emergency medical systems (HEMS). The STEMI patients from Galati are transferred to either one of 2 PCI centers, Iasi or Bucharest, cities located at 240 km (Bucharest) and 226 km respectively (Iasi). To date, it has not been established whether the transfer to a certain center is more efficient or whether there are significant time differences that would necessitate the transfer of the STEMI patient from Galati exclusively to Iasi or Bucharest. Regarding interhospital transfers executed either through HEMS or GEMS for STEMI patients, various studies in the United States and Europe have brought up controversial results, and they were limited in scope with a small series of patients [9,10,11,12,13,14,15,16]. The current study is the first one in Romania that evaluates the air and ground transfer of STEMI patients to PCI centers.

This study aimed to assess the delays that can potentially occur in the emergency transfer of patients with ST-elevation myocardial infarction (STEMI) to percutaneous coronary intervention (PCI) centers when comparing the transportation methods used, in the south-east region of Romania which does not have a PCI center.

## 2. Materials and Methods

### 2.1. Study Population and Study Protocol

SMURD (Mobile Emergency Service, Resuscitation and Extraction Service) is an integrated public intervention unit without jurisdiction, encompassing great strategic importance for Romania. Air transfer is used when the proper transfer cannot be provided by land or if the transfer time by land is longer than the patient’s condition allows, causing further and/or irreversible complications. The SMURD Galati air unit (HEMS Galati) can perform primary, secondary and special missions. In the current study, we evaluated the activity of an HEMS Galati unit for secondary missions only, which are patient transfer missions from medical care units unable to provide adequate investigation capacity or medical care and require transfer to a unit with PCI facilities.

We conducted a retrospective study by evaluating the medical documents of about 97 patients who were admitted to the UPU Galati (Emergency Department), during the year 2018 with the diagnosis of STEMI and meeting eligibility criteria for PCI, warranting transfer to more specialized centers. The analysis was performed on subgroups of patients diagnosed with STEMI who benefited from either air transfer (HEMS group) or ground transfer (GEMS group) between 1 January 2018 and 31 December 2018. Out of the 97 patients included in the study, 52 patients (53.61%) were transferred by land to the assigned PCI centers: 31 patients (31.96%) were transferred to Iasi, while 21 patients (21.65%) were transferred to Bucharest. The remaining 45 patients in the study (46.39%) were transported by air to specialized centers: 30 patients (30.93%) were transported by air to a PCI center in Bucharest and 15 patients (15.46%) to a PCI center in Iasi.

Regarding the activity of HEMS Galati, throughout the year of 2018, it performed a total of 121 secondary missions for patients diagnosed with STEMI, but only 45 flights (37.19%) represented the air transport of STEMI patients to PCI units, the rest of the patients requiring air transportation being: STEMI patients with a timeframe from the onset of symptoms outside the first 24 h from the debut, patients who were not eligible for PCI or patients diagnosed with STEMI during hospitalization for pathologies other than STEMI at the Galati County Emergency Clinical Hospital.

The criteria used for inclusion in the study were the age 18 or higher, the STEMI diagnosis confirmed by electrocardiogram at the admission time with an ST segment elevation higher than 1 mm (>1 mV) or a new or presumed new left branch block (according to diagnostic criteria specified in guidelines of the European Society of Cardiology) [4], patients meeting eligibility criteria for PCI. 

Data collection was designed to accurately highlight the patients’ traits including: (1) relevant medical history information (e.g., cardiovascular risk factors such as obesity, smoking, dyslipidaemia, etc.); (2) important features of the clinical evaluation during admission (assessment of STEMI patients according to Killip classification); and (3) the proper times of the reperfusion process (onset of symptoms, first medical contact in the Emergency Department, first ECG showing ST segment elevation, patient pick-up time for transfer as well as the duration of air or ground transport to the assigned PCI unit). The medical information collected constitutes part of the medical documentation filled out at the time of patient’s admission at the emergency department. The study conducted is observational only and did not involve any further documentation or interventions other than standard care and usual medical records. The institution’s ethics council processed our request for following through with the current study and gave a favorable review.

As for the medical crew that ensured the patients’ transfer to the PCI centers, both by air and by land, they used a similar layout—doctor and nurse, both means of transportation using similar equipment. STEMI patients transferred by land to PCI centers with ambulances in the presence of a medical crew consisting of nurses only were excluded from the study. The choice of HEMS or GEMS depended entirely on the availability of the crew, weather conditions and time of day, given that our HEMS operate only during the day. HEMS helicopters have a top speed of 250 km/h, while GEMS, using ambulances, can achieve a maximum speed of 130 km/h on highways and 80 km/h on common roadways.

### 2.2. Data Analysis

Continuous data (variables) were expressed as the mean +/− standard deviation (SD) or median and IQR (interquartile range). Student’s *t*-test or Mann–Whitney test, Chi-Square test or Fisher’s Exact test were chosen to test differences in continuous respectively categorical data between groups (HEMS vs. GEMS), when appropriate [17,18,19]. The results of the normal distribution analysis were obtained using the Kolmogorov–Smirnov test, for the following data: age, transportation times T1 and T2 (asymp. sig. 2-sided test < 0.05). A *p* value < 0.05 is considered statistically significant. 

## 3. Results

The main characteristics of the patients enrolled in the study are presented in Table 1. Patients eligible for PCI at the time of transfer were subsequently divided into two groups, depending on the transfer medium chosen: air or land. Patients were also evaluated according to the response time to the request for transfer (T1) as well as the actual transfer time (T2). The processing time for patients in the emergency unit (T1), as well as the patient transfer time to PCI centers in Iași or Bucharest (T2), accurately reflects the on-site events, these measurements being recorded electronically in real-time by the HEMS and GEMS teams which were able to provide the data for this study. 

Assessing the average age of the patients in the study, it is noted that female patients had a significantly higher mean age compared to male patients in both the group of air transportation patients (70.82 ± 7.5 years vs. 62.21 ± 10.22 years, *p* = 0.006) as well as by land (71 ± 8.56 years vs. 60 ± 14.17 years, *p* = 0.001). It is worth noting that the study enrolled significantly more male patients (70 patients) than female patients (27 patients, *p* < 0.05).

**Figure 1:**
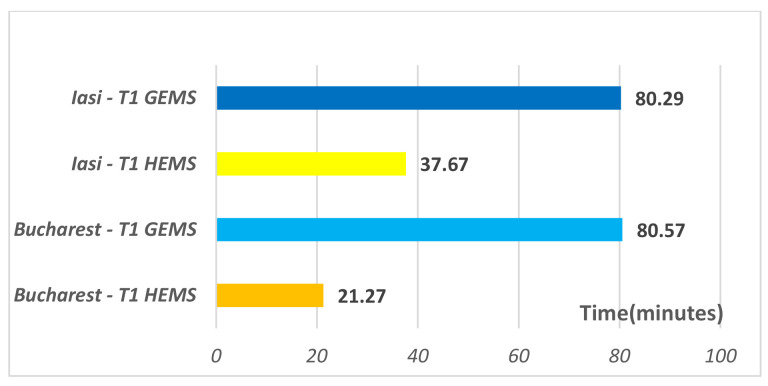
Response time (T1) when taking the patient from the emergency department for transfer by air or land.

**Figure 2:**
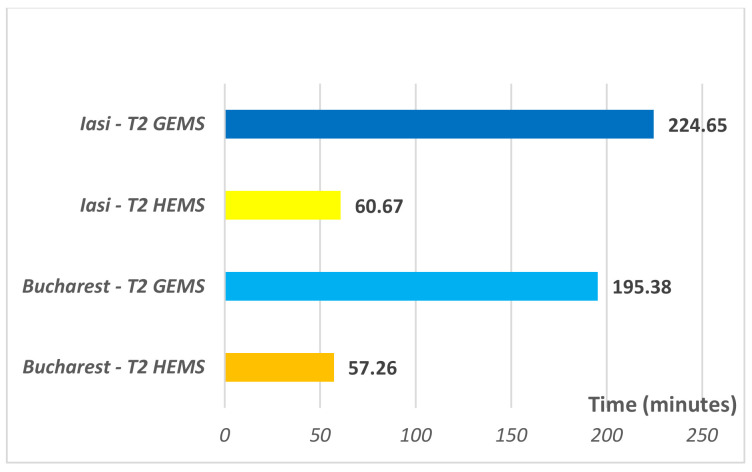
Effective air and ground transfer time (T2) to PCI centers.

As for the patients’ processing time from the emergency department by the transfer crew (T1), it is significantly lower *(p* < 0.05) for patients who benefited from air transfer (Table 2), regardless of the PCI center to where the transfer was made, Iasi or Bucharest (Figure 1). This could be explained by a more thorough selection of patients eligible for air transfer in the dispatcher’s office (comas, stroke, trauma, STEMI) when compared to ground teams which have the commitment to respond to all requests made to the emergency number 112. Moreover, both a nurse and a doctor are mandatory for air transfer, while for ground transportation it is oftentimes necessary to wait for the full crew to form (nurse and doctor), who may be involved in responding to other emergencies. Specialty literature does not provide specifics regarding the ambulance crews’ reaction times to the requests made from the emergency departments for the transfer of patients to a PCI center, either by air or land. The T2 transfer time represents the time spent while travelling by air or by ground ambulance to PCI centers, and the differences between the two ways of transportation are statistically significant (*p* < 0.000). The air transfer is at least three times faster than its land counterpart (Figure 2). Even with a much lower timeframe ensured by air transportation, only 80% of STEMI patients transferred by HEMS arrived at the PCI centers in the target range for PCI specified by the therapeutic guidelines, namely only those patients that did not encounter any delays in processing them from the admission area. No ground transferred patients reached PCI centers in less than 120 min.

## 4. Discussion

Studies in Europe and the United States (USA) show that use of HEMS can allow STEMI patients in remote regions to achieve similar results to patients who are admitted directly to a PCI specialized center, the results being better than those obtained by thrombolytic therapy in reference hospitals [20,21]. In our study, only patients transferred by HEMS to PCI centers were able to benefit from a treatment similar to those who went directly to the PCI center in Iasi or Bucharest. 

Based on most relevant estimates in the literature [22,23], for the time savings to be clinically significant they need to account for at least 15 min. More specifically, HEMS has to achieve at least 15 min of total time savings to be defined as clinically relevant. According to similar studies, time savings would translate into an estimate known as “lives saved per 100 transport”, patients whose deaths would be averted taking into account solely the time savings obtained during transportation [24,25]. In our study, the HEMS time savings are clinically important and they derive from both the reduction of the time needed to process the patient from the emergency department (savings of at least 42.62 min) and the time savings resulting from the transfer time, the air transportation being significantly faster than the land transportation (time savings accounting for at least 138.12 min).

Other studies showing that HEMS reduces transportation time compared to GEMS, but does not significantly contribute to patients’ access to PCI within 120 min as recommended. In these studies, transportation using HEMS proved insufficient to access PCI early [26]. Conversely, in our study, 80% of air transported patients had access to PCI in less than 120 min. 

Taking the distance to the PCI center into consideration, studies have shown that helicopter transportation of STEMI patients was five times less efficient than ground transportation in delivering the patient within the initial 90-min window from the first medical contact to the percutaneous coronary intervention-capable facility recommended by the guidelines, especially for transfer distances of 50 km or less [16]. That implies if the distance to the PCI center is over 50 km, it may be more preferable for the transfer to be executed by air, as was the case of our study (the distance to any of the 2 PCI centers is over 220 km), which resulted in 64.44% of patients transferred by air reaching the target range recommended by the guidelines of 90 min or less, while 15.56% of patients had a transfer time between 90 to 120 min. The rest of the patients (20%) transferred by air reached the PCI center with a total transfer time of 120 min or more, due to delays in processing the patient (T1) from the Emergency Department. 

Additionally, in the current study, we tested whether either of the 2 PCI centers (Iași or Bucharest) provide a significant time saving when transferring patients, considering that the centers are situated at relatively similar distances (Table 1). By analyzing the patient transfer times by air (HEMS) to either Iași or Bucharest, we could not establish a significant difference in either the patients’ processing—T1 (Bucharest—8(7–45) min; Iasi—44(5–67) min, *p* = 0.088) or the T2 transfer time (Bucharest—57.50(54–60) min; Iasi—60(55–65) min, *p* = 0.133). Conversely, when terrestrial routes are taken into account, even if we could not establish a significant difference in patient processing time—T1 (*p* > 0.05), the transfer time to the Bucharest PCI center is markedly shorter compared to the Iași PCI center (Bucharest—195.38 ± 30.42 min; Iasi—224.65 ± 28.48 min, *p* = 0.001). Considering this, when a patient is to be transferred using terrestrial routes, the Emergency Department of the County Emergency Hospital Galați strongly recommends the Bucharest PCI center as the first option, which can provide on average a 29-min saving, improving the patients’ accessibility to the PCI procedures.

The safety of patients transported by air was also a concern for previous studies which have provided conflicting results. Topol et al. [27] and Kaplan et al. [28] suggested that the transportation of patients with acute myocardial infarction by helicopter was safe. In contrast, Schneider et al. [29] reported that the incidence of cardiogenic shock, bradycardia, arrhythmia, chest pain and seizures was more common in patients transported by air ambulance (41%) compared to those transported by ground ambulance (7.5%). Tyson et al. [30] found serum catecholamine concentrations to be higher in patients transported by air than in those transferred by land which can contribute to the development of arrhythmias. Stone et al. [31] reported that interfacility transportation of cardiac patients by air ambulance does not offer any advantage over ground ambulance transport. The air-transported patients in our study had permanent EKG monitoring during transportation and there were no cardiac arrhythmias or other complications to report during the transfer.

Therefore, for STEMI patients admitted to non-PCI hospitals, there is an urgent need for a systemic approach to distinguish between patients that can be transferred to a PCI center with adequate door-to-device time and those whose transfer can be postponed, arriving at the PCI center outside the critical first 120 min. This differentiation is difficult to achieve in practice because medical professionals do not possess an instrument capable of estimating the total delay the patient will encounter from the first medical contact. Thus, more PCI centers at shorter distances are required to provide similar transfer times when choosing either transportation method, GEMS or HEMS. 

Furthermore, for STEMI patients who present to non-PCI centers outside a reasonable range from PCI centers, it is necessary to emphasize the role of fibrinolytic therapy administered promptly (in the absence of contraindications), in less than 10 min from the diagnosis confirmation [4].

### Limitations

This is a unicentric study covering a confined area of the country and a small patient sample was used. Our results and recommendations may not be relevant across the entire country area, their applicability requiring extensive studies to streamline the medical system and reduce intra- and interfacility transfer times. Moreover, some cases of STEMI can be challenging on a technical level pertaining to non-medical events, and delays can occur due to inability to obtain the medical consent from either the patient or his family, abnormal laboratory findings or misdiagnosis.

## 5. Conclusions

In a well-developed public healthcare system, the STEMI treatment relies heavily on adequate circuits, both intra- and interfacility, in order to achieve an acceptable timeframe that complies with the guidelines’ recommendation of 120 min or less, which can pose a significant challenge, especially in those cases that require an inter-hospital transfer. 

In order to reduce the primary intervention time for STEMI patients, an ample nation-wide healthcare policy is required with the following objectives: (1) increasing the general population awareness of the STEMI disease and its symptoms with prompt admission in the closest medical unit; (2) HEMS logistics improvement by increasing the available number of helicopters capable of also performing night flights and thus all STEMI patients to benefit from a sub-120 min processing time before arriving at the PCI center. Furthermore, if non-PCI centers are further away from PCI centers, terrestrial transportation means should be reserved only for those cases where air transportation becomes unfeasible because of the weather conditions.

## Figures and Tables

**Table 1:** Characteristics of the study population.

Patients Characteristics		HEMS **		GEMS ***		*p*-Value
		Bucharest	Iasi	Bucharest	Iasi	
Distance, km		190	194	240	226	
Nr. of patients, no * (%)		30 (30.93%)	15 (15.46%)	21 (21.65%)	31 (31.96%)	0.543 ^a^
Age (M ± SD)		63.63 ± 8.07	65.67 ± 13.83	61.90 ± 15.53	64 ± 12.39	0.659 ^b^
Sex, no* (%)	Male	24 (80.00%)	10 (66.66%)	13 (61.90%)	23 (74.19%)	0.488 ^c^
	Female	6 (20.00%)	5 (33.33%)	8 (38.09%)	8 (25.80%)	
Environment, no * (%)	Urban	32 (71.11%)		35 (67.30%)		0.686 ^c^
	Rural	13 (28.89%)		17 (32.69%)		
Fibrinolysis, no * (% of 97)	YES	25 (25.77%)	6 (6.19%)	18 (18.56%)	2 (2.06%)	0.003 ^c^
	NO	5 (5.15%)	9 (9.28%)	3 (3.09%)	29 (29.90%)	
Comorbidities, no * (% of 97)	Current smoker	4 (4.12%)	1 (1.03%)	1 (1.03%)	1 (1.03%)	0.244 ^d^
	Ischemic heart disease	5 (5.15%)	4 (4.12%)	5 (5.15%)	4 (4.12%)	0.734 ^c^
	Coronary angioplasty in the past	1 (1.03%)	0 (0%)	0 (0%)	0 (0%)	0.464 ^d^
	Hypertension	13 (13.40%)	4 (4.12%)	6 (6.19%)	11 (11.34%)	0.601 ^c^
	Acute pulmonary oedema	1 (1.03%)	1 (1.03%)	0 (0%)	0 (0%)	0.213 ^d^
	Obesity	9 (9.28%)	5 (5.15%)	1 (1.03%)	3 (3.09%)	0.003 ^c^
	Atrial Fibrillation	1 (1.03%)	0 (0%)	0 (0%)	2 (2.06%)	1.000 ^d^
	Neoplastic diseases	2 (2.06%)	0 (0%)	0 (0%)	0 (0%)	0.213 ^d^
	Diabetes	2 (2.06%)	2 (2.06%)	3 (3.09%)	5 (5.15%)	0.333 ^c^
	Dyslipidemia	10 (10.31%)	6 (6.19%)	3 (3.09%)	9 (9.28%)	0.176 ^c^
	Permanent cardiostimulation	0 (0%)	1 (1.03%)	0 (0%)	0 (0%)	0.464 ^d^
	Atrioventricular block grade III	0 (0%)	1 (1.03%)	0 (0%)	2 (2.06%)	1.000 ^d^
	Chronic kidney disease	0 (0%)	0 (0%)	1 (1.03%)	3 (3.09%)	0.121 ^d^
	Ischemia localization, no * (% of 97)	Anteroseptal	5 (5.15%)	0 (0%)	3 (3.09%)	3 (3.09%)	0.947 ^c^
		Inferior	4 (4.12%)	1 (1.03%)	2 (2.06%)	8 (8.25%)	0.386 ^c^
		Infero-posterior	2 (2.06%)	3 (3.09%)	4 (4.12%)	2 (2.06%)	0.947 ^c^
Infero-lateral		10 (10.31%)	5 (5.15%)	3 (3.09%)	5 (5.15%)	0.038 ^c^
Anterior		7 (7.22%)	5 (5.15%)	7 (7.22%)	11 (11.34%)	0.398 ^c^
Postero-lateral		2 (2.06%)	1 (1.03%)	2 (2.06%)	2 (2.06%)	1.000 ^d^
Killip classification, no * (% of 97)	Killip I	20 (20.62%)	8 (8.25%)	12 (12.37%)	19 (19.59%)	0.982 ^d^
	Killip II	7 (7.22%)	4 (4.12%)	5 (5.15%)	9 (9.28%)	
	Killip III	0 (0%)	3 (3.09%)	1 (1.03%)	2 (2.06%)	
	Killip IV	3 (3.09%)	0 (0%)	3 (3.09%)	1 (1.03%)	

* no—number of patients, ** GEMS—Ground Emergency Medical Services, *** HEMS—Helicopter Emergency Medical Services, M—mean, SD—standard deviation, the *p*-value was obtained with: ^a^—binomial test, ^b^—Student *t* test, ^c^—Chi-Square test, ^d^—Fisher’s Exact test.

**Table 2:** Comparative analysis of the GEMS and HEMS transfer times of STEMI patients to Bucharest and Iasi PCI centers.

PCI Center	T1 (min)	T2 (min)	Comparative Analysis	T1	T2
**B-HEMS**	8(7–45)	57.50(54–60)	*p* (B-HEMS vs. GEMS)	0.000 ^a^	0.000 ^b^
	21.27 ± 20.42	57.26 ± 4.29			
**B-GEMS**	56(38–83)	191(185–201)			
	80.57 ± 80.78	195.38 ± 30.42			
**IS-HEM**S	44(5–67)	60(55–65)	*p* (IS-HEMS vs. GEMS)	0.004 ^a^	0.000 ^b^
	37.67 ± 32.35	60.67 ± 7.80			
**IS-GEMS**	73(47–93)	222(205–248)			
	80.29 ± 65.55	224.65 ± 28.48			
**HEMS**	8(7–53)	58(54–62)	*p* (HEMS vs. GEMS)	0.000 ^a^	0.000 ^b^
	26.73 ± 25.86	58.40 ± 5.84			
**GEMS**	71.50(38.50–89.50)	207.50(192.50–234.50)			
	80.40 ± 71.32	212.83 ± 32.40			

B—Bucharest PCI center, IS—Iasi PCI center, the *p*-value was obtained with: ^a^—Mann–Whitney test, ^b^—Student *t* test.

## Data Availability

Any further information concerning the case study is available from the corresponding author upon reasonable request.
